# Biomechanics and Oxidative Stress: An Integrative Review of the Redox–Mechanobiological Axis and the Role of Trace Elements in Disease

**DOI:** 10.1007/s12011-026-05163-w

**Published:** 2026-05-25

**Authors:** Servet Kavak

**Affiliations:** Department of Biophysics, Faculty of Medicine, Izmir Bakircay University, Izmir, Turkey

**Keywords:** Oxidative stress, Mechanobiology, Biomechanics, Reactive oxygen species, Mechanotransduction, YAP/TAZ, Focal adhesion kinase, Cytoskeletal remodeling, Mitochondrial dysfunction, Endothelial stiffness, Ferroptosis, Trace elements, Selenium, Zinc, Iron, COVID-19

## Abstract

Oxidative stress and mechanobiological signaling are increasingly recognized as interconnected determinants of cellular and systemic homeostasis. Reactive oxygen species (ROS), traditionally associated with molecular damage, are now understood to directly regulate cytoskeletal remodeling, membrane viscoelasticity, mitochondrial dynamics, and mechanotransduction pathways including focal adhesion kinase (FAK), integrins, and Yes-associated protein/transcriptional coactivator with PDZ-binding motif (YAP/TAZ). Conversely, biomechanical forces such as extracellular matrix stiffness, cyclic stretch, and disturbed shear stress modulate intracellular redox signaling through mitochondrial dysfunction, NADPH oxidase activation, and inflammatory pathways. This bidirectional interaction forms a self-amplifying “redox–mechanobiological axis” that contributes to endothelial dysfunction, fibrosis, thrombosis, tumor progression, and viral pathophysiology.Trace elements, particularly selenium, zinc, and iron, emerge as critical modulators of this axis by influencing antioxidant defense systems, cytoskeletal integrity, membrane stability, ferroptosis, and cellular stiffness. Selenium-dependent selenoproteins regulate mitochondrial redox balance and actin organization; zinc stabilizes membrane architecture and mechanosensitive proteins; and iron-mediated Fenton chemistry promotes oxidative injury, ferroptosis, and biomechanical alterations.This review integrates molecular mechanisms, mechanobiological principles, and recent clinical findings—particularly from cardiovascular disease, cancer biology, and COVID-19—to propose a unified framework linking oxidative stress to biomechanical dysfunction. In addition, current controversies, methodological limitations, and future therapeutic directions targeting the redox–mechanobiological axis are critically discussed.

## Introduction

Oxidative stress is classically defined as an imbalance between reactive oxygen species (ROS) production and antioxidant defense systems, leading to oxidative modification of lipids, proteins, and nucleic acids [1, 2]. Although traditionally investigated as a biochemical phenomenon, increasing evidence demonstrates that oxidative stress also exerts profound effects on cellular and tissue biomechanics [3, 4].

Biomechanics governs cellular stiffness, elasticity, deformability, and mechanotransduction through coordinated interactions among the cytoskeleton, extracellular matrix (ECM), focal adhesion complexes, and plasma membrane architecture [5–7]. Mechanical forces such as shear stress, cyclic stretch, and matrix rigidity dynamically regulate intracellular signaling pathways that determine cell survival, migration, inflammation, and differentiation [6–8].

Recent studies indicate that ROS directly regulate actin polymerization, microtubule stability, focal adhesion turnover, and mechanosensitive signaling pathways including integrin–FAK signaling and YAP/TAZ activation [9–12]. Oxidative modification of cytoskeletal proteins alters cellular viscoelasticity and membrane deformability, while mitochondrial ROS production influences mechanotransduction through ATP depletion and calcium dysregulation [13, 14]. Conversely, biomechanical stress itself modulates ROS generation through mitochondrial deformation, NADPH oxidase activation, and inflammatory signaling cascades [15, 16]. These findings support the concept of a bidirectional “redox–mechanobiological axis,” in which oxidative stress and biomechanical dysfunction continuously amplify one another.

Trace elements are increasingly recognized as essential regulators of this axis. Selenium-dependent antioxidant enzymes maintain mitochondrial redox balance and cytoskeletal stability; zinc contributes to membrane integrity and mechanosensitive protein regulation; and iron dysregulation promotes ROS generation, ferroptosis, and increased cellular stiffness through Fenton chemistry [17–21]. Dysregulation of these micronutrients has been associated with cardiovascular disease, cancer progression, fibrosis, and COVID-19 severity.

The aim of this review is to integrate current evidence regarding oxidative stress, mechanobiology, and trace element regulation into a unified mechanistic framework. Particular emphasis is placed on ROS-mediated cytoskeletal remodeling, mitochondrial–cytoskeletal crosstalk, endothelial biomechanics, mechanotransduction signaling, and disease-specific implications of the redox–mechanobiological axis.

## Molecular Basis of Oxidative Stress

Reactive oxygen species, including superoxide anion, hydrogen peroxide, and hydroxyl radicals, are primarily generated in mitochondria during oxidative phosphorylation [1, 15]. At physiological levels, ROS function as signaling molecules regulating cellular processes. However, excessive ROS leads to oxidative damage of lipids, proteins, and DNA [2].

Mitochondrial dysfunction plays a central role in ROS amplification through disruption of electron transport chain activity, mitochondrial membrane depolarization, calcium dysregulation, and excessive superoxide generation, thereby establishing a self-perpetuating oxidative feedback cycle [16, 37].

Schematic representation of the redox–mechanobiological axis illustrating the bidirectional interaction between oxidative stress and cellular biomechanics. Reactive oxygen species (ROS), derived from endogenous sources (mitochondria, NADPH oxidase, xanthine oxidase) and exogenous stimuli (inflammation, infection, radiation, hazardous chemical exposure, and mechanical stress), induce cytoskeletal damage through actin oxidation and stress fiber remodeling [18, 24], membrane lipid peroxidation via ROS-mediated phospholipid oxidation [20, 21], endothelial dysfunction associated with nitric oxide depletion and vascular inflammation [22, 23], and mitochondrial impairment characterized by membrane depolarization and impaired oxidative phosphorylation [16, 37].

These alterations result in increased cellular stiffness, reduced deformability, and impaired mechanotransduction, collectively leading to altered cellular and tissue mechanics. Mechanical stress conditions, including shear stress, stretch, and extracellular matrix stiffness, further exacerbate mitochondrial dysfunction and ROS production.

**Fig. 1:**
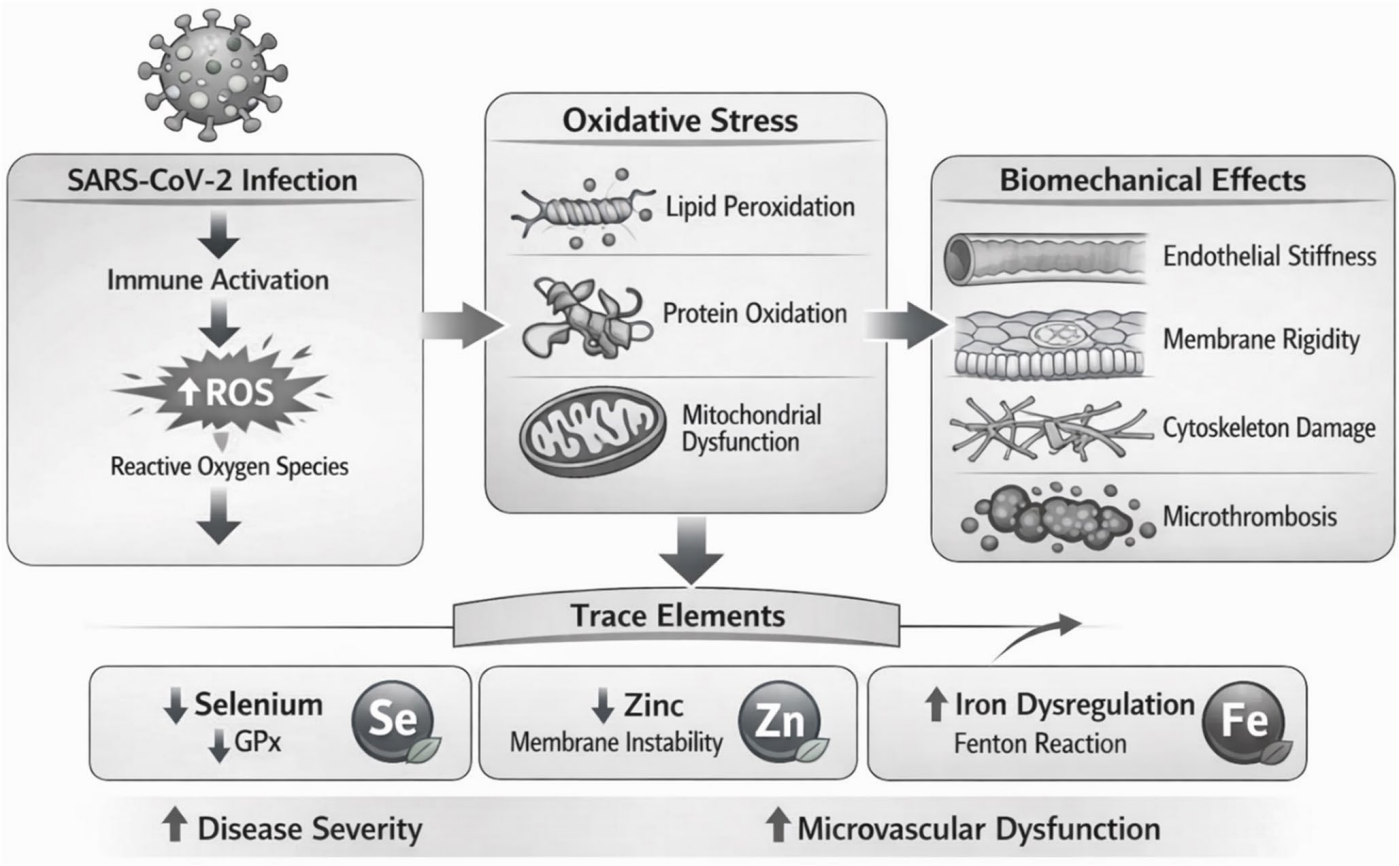
Redox Imbalance, Trace Element Dysregulation, and Biomechanical Dysfunction in COVID-19. Schematic representation of SARS-CoV-2–induced oxidative stress and its association with trace element imbalance and biomechanical dysfunction. Viral infection triggers immune activation and excessive generation of reactive oxygen species (ROS), leading to lipid peroxidation, protein oxidation, and mitochondrial dysfunction. These processes contribute to endothelial stiffness, membrane rigidity, cytoskeletal damage, and microthrombosis. Alterations in trace elements, including decreased selenium and zinc levels and increased iron-mediated redox activity, further exacerbate oxidative stress, thereby promoting microvascular dysfunction and increased disease severity

The integration of these processes establishes a redox–mechanical feedback loop, in which biomechanical alterations amplify oxidative stress, driving disease progression. Downstream consequences include cardiovascular disorders, fibrosis, cancer progression, and viral infections such as COVID-19. Figure 1.

## Molecular Mechanisms Linking Oxidative Stress and Mechanobiology

ROS induce oxidative modifications of cytoskeletal proteins, particularly actin, leading to structural reorganization and increased cellular stiffness [17, 18]. These changes impair cellular deformability and disrupt microcirculation [19].

Lipid peroxidation further alters membrane biomechanics by reducing fluidity and increasing rigidity, thereby affecting cellular signaling and transport processes [20, 21].

**Fig. 2:**
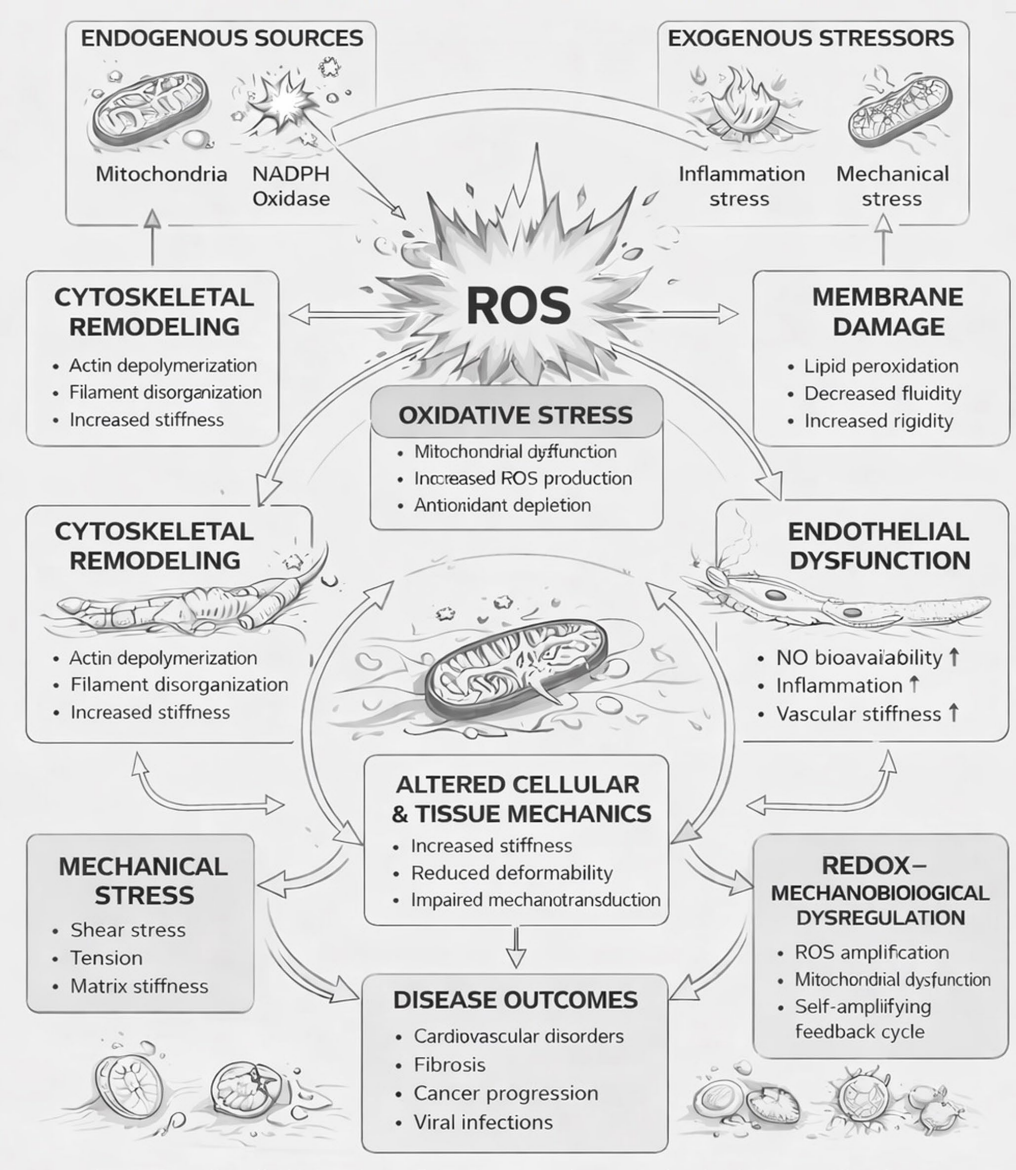
The Redox–Mechanobiological Axis in Cellular Dysfunction. Schematic illustration of the bidirectional interplay between oxidative stress and mechanobiological processes in cellular dysfunction. Endogenous sources, including mitochondria and NADPH oxidase, together with exogenous stressors such as inflammation and mechanical stress, promote excessive production of reactive oxygen species (ROS). Elevated ROS levels induce oxidative stress, characterized by mitochondrial dysfunction, increased ROS generation, and antioxidant depletion. These changes lead to cytoskeletal remodeling, membrane damage, and endothelial dysfunction, resulting in altered cellular and tissue mechanics, including increased stiffness, reduced deformability, and impaired mechanotransduction. The persistent interaction between redox imbalance and biomechanical alterations establishes a self-amplifying cycle, ultimately contributing to disease outcomes such as cardiovascular disorders, fibrosis, cancer progression, and viral infections

Endothelial cells are particularly sensitive to oxidative stress. ROS reduce nitric oxide (NO) bioavailability, resulting in vasoconstriction, inflammation, and vascular stiffness [22, 23]. These changes contribute to microvascular dysfunction and thrombosis. Figure 2.

### ROS-Mediated Cytoskeletal Remodeling

The cytoskeleton is highly sensitive to oxidative modifications and represents one of the principal targets through which ROS alter cellular biomechanics. Reactive oxygen species directly oxidize actin thiol groups, disrupt actin polymerization dynamics, and alter microtubule stability, leading to impaired cellular deformability and increased stiffness [22, 23]. Oxidative stress additionally activates RhoA/Rho-associated kinase (ROCK) signaling, promoting actomyosin contractility and stress fiber formation [24].

Oxidative modifications of cytoskeletal proteins are particularly relevant in endothelial cells and erythrocytes, where altered membrane deformability contributes to microvascular dysfunction and impaired perfusion [25]. Experimental studies using atomic force microscopy have demonstrated that ROS exposure significantly increases endothelial stiffness and reduces cellular elasticity [26].

Mitochondrial dysfunction further amplifies these biomechanical alterations. Increased mitochondrial ROS production impairs ATP generation and calcium homeostasis, both of which are essential for cytoskeletal dynamics and focal adhesion turnover [27]. Consequently, oxidative stress establishes a self-reinforcing cycle in which mitochondrial dysfunction and cytoskeletal remodeling perpetuate one another.

### Mitochondrial–Cytoskeletal Crosstalk

Bidirectional communication between mitochondria and the cytoskeleton is increasingly recognized as a central determinant of cellular mechanobiology. Microtubules and actin filaments regulate mitochondrial positioning, fission–fusion dynamics, and intracellular trafficking [28]. In turn, mitochondrial metabolism and ROS generation regulate cytoskeletal organization through redox-sensitive signaling pathways.

Excessive ROS disrupt mitochondrial membrane potential and activate mitochondrial permeability transition pathways, resulting in calcium dysregulation and altered actomyosin mechanics [29]. Furthermore, oxidative stress-induced mitochondrial fragmentation has been linked to increased cellular stiffness, impaired migration, and endothelial dysfunction [30].

This interaction is particularly important under conditions of mechanical stress. Shear stress and matrix stiffness alter mitochondrial morphology and bioenergetic function, thereby modulating ROS generation and inflammatory signaling [31]. These findings highlight mitochondrial–cytoskeletal crosstalk as a critical component of the redox–mechanobiological axis.

### Integrin, FAK, and YAP/TAZ Mechanotransduction Signaling

Mechanotransduction pathways are strongly influenced by oxidative stress. Integrins function as mechanosensitive receptors that transmit extracellular mechanical cues into intracellular biochemical responses through focal adhesion kinase (FAK) activation [32]. ROS modulate integrin clustering and FAK phosphorylation, thereby altering cytoskeletal organization, cell adhesion, and migration.

Increased matrix stiffness and oxidative stress also activate YAP/TAZ signaling pathways, which regulate cellular proliferation, fibrosis, and inflammatory responses [33]. Persistent YAP/TAZ activation has been implicated in vascular remodeling, tumor progression, and fibrotic diseases [34].

Importantly, ROS-mediated YAP/TAZ activation appears to be closely linked to mitochondrial dysfunction and altered cytoskeletal tension. This establishes an integrated mechanoredox signaling network in which oxidative stress and mechanical forces cooperatively regulate disease progression.

## Biomechanical Regulation of Redox Homeostasis

Mechanical forces regulate ROS production and antioxidant responses. Laminar shear stress promotes endothelial homeostasis, whereas disturbed flow increases oxidative stress and inflammation [24, 25].

The cytoskeleton plays a key role in mitochondrial positioning and function. Disruption of cytoskeletal integrity impairs mitochondrial dynamics, leading to increased ROS production [26].

This establishes a self-amplifying feedback loop in which oxidative stress alters cytoskeletal organization, mitochondrial bioenergetics, and membrane mechanics, while biomechanical abnormalities such as matrix stiffness, shear stress disturbances, and altered actomyosin tension further increase mitochondrial ROS production and NADPH oxidase activation. Persistent mechanoredox signaling may therefore promote chronic inflammation, endothelial dysfunction, fibrosis, and progressive tissue remodeling.

Schematic illustration of oxidative stress–mediated biomechanical dysfunction in COVID-19. SARS-CoV-2 infection triggers immune activation and excessive production of reactive oxygen species (ROS), leading to oxidative damage, including lipid peroxidation, protein oxidation, and mitochondrial dysfunction.

These molecular alterations result in biomechanical changes such as endothelial stiffness, membrane rigidity, cytoskeletal disruption, and microthrombosis. Trace elements play a critical modulatory role in this process. Selenium deficiency impairs antioxidant enzyme activity, particularly glutathione peroxidase, enhancing oxidative stress. Zinc deficiency contributes to membrane instability and impaired immune responses. Iron dysregulation promotes ROS generation through Fenton reactions, further amplifying oxidative damage.

Collectively, these mechanisms contribute to increased disease severity and microvascular dysfunction, highlighting the importance of micronutrient homeostasis in the redox–mechanobiological axis. Figure 2.

## Trace Elements and the Redox–Mechanobiological Axis

Trace elements are not only biochemical cofactors but also critical regulators of cellular biomechanics, membrane architecture, mitochondrial integrity, and mechanotransduction signaling.

### Selenium

Selenium is an essential component of antioxidant enzymes such as glutathione peroxidase. Selenium deficiency impairs ROS detoxification and increases oxidative damage [27, 28]. Selenium is an essential component of multiple antioxidant selenoproteins, including glutathione peroxidases (GPXs) and thioredoxin reductases, which regulate mitochondrial redox homeostasis and limit ROS-mediated damage [35, 36]. Emerging evidence suggests that selenium also influences cytoskeletal organization and cellular biomechanics. GPX-dependent regulation of intracellular ROS preserves actin filament integrity and prevents oxidative disruption of focal adhesion complexes [37]. Selenium deficiency has been associated with impaired endothelial elasticity, mitochondrial dysfunction, and increased inflammatory stiffness responses [38].

In addition, selenium may modulate mechanotransduction indirectly through mitochondrial protection. By preserving mitochondrial membrane potential and ATP production, selenium supports cytoskeletal dynamics and cellular deformability. Experimental studies further suggest that selenium supplementation attenuates endothelial dysfunction and fibrosis through inhibition of ROS-sensitive TGF-β and YAP/TAZ signaling pathways [39].

### Zinc

Zinc regulates immune function and membrane stability. Zinc deficiency increases oxidative stress and disrupts cellular integrity [29, 30]. Zinc plays fundamental roles in antioxidant defense, membrane stabilization, and immune regulation [40, 41]. Zinc-dependent proteins regulate cytoskeletal assembly, tight junction integrity, and mechanosensitive ion channels. inc deficiency disrupts membrane phospholipid stability and increases susceptibility to lipid peroxidation, resulting in altered membrane fluidity and increased rigidity [42]. Zinc additionally modulates actin polymerization and focal adhesion dynamics through zinc-finger proteins and metalloproteinase regulation [43].

At the mechanobiological level, zinc deficiency has been associated with impaired endothelial barrier function, altered cell migration, and increased inflammatory stiffness responses. Moreover, zinc participates in the regulation of mechanosensitive transcription factors and antioxidant signaling pathways, including NF-κB and Nrf2 [44]. These observations support the concept that zinc contributes to both redox regulation and biomechanical homeostasis.

### Iron

Iron dysregulation promotes ROS production via Fenton reactions, amplifying oxidative damage and contributing to cellular stiffness [31]. Iron is essential for mitochondrial respiration and oxygen transport; however, excess free iron promotes ROS generation through Fenton chemistry [45]. Iron-mediated oxidative stress contributes directly to biomechanical dysfunction by inducing lipid peroxidation, ferroptosis, and cytoskeletal damage [46]. Increased intracellular iron has been associated with enhanced cellular stiffness, impaired endothelial deformability, and extracellular matrix remodeling [47].

Ferroptosis, a form of iron-dependent regulated cell death characterized by lipid peroxidation and membrane destabilization, has recently emerged as a major contributor to vascular injury, fibrosis, and cancer progression [48]. Iron dysregulation also affects mitochondrial mechanics and ATP production, thereby influencing mechanotransduction and cytoskeletal organization.

Collectively, selenium, zinc, and iron act as integrative modulators of the redox–mechanobiological axis by simultaneously influencing oxidative stress, membrane mechanics, cytoskeletal stability, and mitochondrial function.

## Clinical Evidence and Integration of Recent Studies

Clinical studies strongly support the role of trace elements in oxidative stress and disease progression.

Özdemir et al. evaluated oxidative stress markers together with selenium and zinc levels following COVID-19 vaccination and observed numerical increases in selenium and zinc concentrations; however, these differences did not reach statistical significance [11]. Nevertheless, the study highlights the dynamic interaction between vaccination-associated immune activation and redox homeostasis [11]. Although COVID-19 represents a clinically important example of systemic oxidative stress and biomechanical dysfunction, similar mechanoredox alterations are also observed in numerous pathological conditions including cardiovascular disease, diabetes mellitus, cancer progression, chronic inflammatory disorders, environmental toxin exposure, ischemia–reperfusion injury, and aging. In these conditions, persistent ROS generation contributes to endothelial stiffening, cytoskeletal remodeling, extracellular matrix dysregulation, mitochondrial dysfunction, and impaired cellular deformability. Therefore, the redox–mechanobiological axis should be considered a generalized pathophysiological framework rather than a mechanism restricted to viral infection alone.

Similarly, oxidative stress and metabolic alterations were observed in COVID-19 patients, indicating a strong relationship between redox status and systemic physiology [12].

Another study demonstrated significant differences in trace elements and electrolytes before and after treatment, suggesting that micronutrient balance influences disease progression [13].

Furthermore, alterations in serum selenium, iron, zinc, and macromineral levels were primarily observed following COVID-19 treatment administered according to Turkish Ministry of Health guidelines [14]. These findings suggest that therapeutic intervention and systemic inflammatory recovery may influence micronutrient homeostasis and oxidative balance [14].

## Discussion

The present review integrates emerging evidence supporting the existence of a dynamic and bidirectional relationship between oxidative stress and cellular biomechanics, conceptualized as the “redox–mechanobiological axis.” Unlike traditional models that examine oxidative stress primarily as a biochemical phenomenon, current evidence indicates that ROS profoundly influence cellular mechanics through cytoskeletal remodeling, mechanotransduction signaling, mitochondrial dysfunction, and membrane viscoelasticity. While oxidative stress has long been characterized as a biochemical imbalance, emerging studies indicate that its impact extends to the mechanical domain, influencing cytoskeletal architecture, membrane viscoelasticity, and cellular deformability [3, 5, 9]. One of the major advances in recent years has been the identification of ROS-sensitive mechanotransduction pathways involving integrins, focal adhesion kinase (FAK), Rho/ROCK signaling, and YAP/TAZ transcriptional regulation. These pathways provide mechanistic explanations for how oxidative stress contributes to endothelial stiffness, fibrosis, tumor invasion, and inflammatory remodeling [32–34]. Simultaneously, biomechanical stress itself increases ROS generation through mitochondrial deformation and NADPH oxidase activation, establishing a self-amplifying feedback cycle.

One of the most consistently reported findings is the effect of reactive oxygen species (ROS) on cytoskeletal remodeling. Oxidative modifications of actin and associated proteins lead to increased cellular stiffness and impaired deformability, as demonstrated in both endothelial cells and erythrocytes [17–19]. These changes are not merely structural but have functional consequences, particularly in microcirculatory flow and tissue perfusion. However, despite strong experimental evidence, the precise molecular pathways linking ROS to cytoskeletal reorganization remain incompletely defined, highlighting an important gap in current knowledge.

Importantly, oxidative stress should not be viewed solely as a biochemical imbalance but also as a biomechanical modifier capable of altering cellular force transmission, membrane viscoelasticity, extracellular matrix interactions, and mechanotransduction signaling. ROS-mediated oxidation of cytoskeletal proteins changes cellular stiffness and deformability, whereas chronic biomechanical stress further exacerbates mitochondrial dysfunction and ROS production. This reciprocal interaction may explain the progressive nature of fibrosis, vascular stiffening, tumor invasion, and chronic inflammatory remodeling observed across multiple disease states.

In parallel, lipid peroxidation has been widely documented as a critical determinant of membrane biomechanics. Oxidative damage to phospholipids reduces membrane fluidity and increases rigidity, thereby impairing mechanotransduction and cellular signaling [20, 21]. While these effects are well established in vitro, their quantitative contribution to disease progression in vivo remains insufficiently characterized. Future studies integrating biophysical measurements with clinical biomarkers are needed to address this limitation.

Endothelial dysfunction represents one of the most clinically relevant manifestations of the redox–mechanobiological axis. ROS-mediated reduction in nitric oxide bioavailability leads to vascular stiffness, inflammation, and pro-thrombotic states [22, 23]. Importantly, disturbed hemodynamic forces further exacerbate oxidative stress, creating a feedback loop in which biomechanical alterations amplify redox imbalance [24, 25]. This interplay is particularly evident in cardiovascular diseases and has been strongly implicated in COVID-19-associated vascular complications.

Recent clinical studies, including those conducted by Özdemir et al., provide valuable insight into the systemic relevance of this axis. Alterations in oxidative stress markers and trace elements such as selenium and zinc following COVID-19 vaccination demonstrate the dynamic nature of redox regulation in humans [11]. Similarly, significant associations between oxidative stress, metabolic parameters, and physical activity levels in COVID-19 patients further support the link between redox balance and systemic physiological function [12].

Moreover, findings showing changes in trace elements and electrolytes before and after COVID-19 treatment suggest that micronutrient homeostasis plays an active role in modulating oxidative stress and potentially cellular mechanics [13, 14]. These observations reinforce the hypothesis that trace elements are integral components of the redox–mechanobiological axis, influencing both molecular and structural aspects of cellular function.

Despite these advances, the current literature remains fragmented. Most studies focus either on molecular mechanisms of oxidative stress or on biomechanical properties of cells, with limited integration between the two. This lack of interdisciplinary research represents a major barrier to fully understanding the complexity of the system. Advanced methodologies, including atomic force microscopy, microfluidic systems, and multi-omics approaches, may help bridge this gap in future investigations.

From a therapeutic perspective, the limited success of antioxidant-based interventions suggests that targeting oxidative stress alone may be insufficient [3, 27]. Given the strong coupling between redox balance and cellular mechanics, effective therapeutic strategies should adopt a dual approach that addresses both biochemical and biomechanical dysfunction. In this context, modulation of cytoskeletal dynamics, improvement of mitochondrial function, and correction of trace element deficiencies represent promising avenues for intervention.

Importantly, the current literature suggests that trace elements are central regulators of this system rather than passive biomarkers of oxidative stress. Selenium-dependent antioxidant enzymes preserve mitochondrial integrity and cytoskeletal organization; zinc regulates membrane stability and mechanosensitive proteins; and iron overload promotes ferroptosis and oxidative stiffening. These findings support a more integrated model in which micronutrient homeostasis directly influences cellular biomechanics.

Trace elements influence nearly every component illustrated in Figs. 1 and 2. Selenium-dependent antioxidant enzymes regulate mitochondrial ROS detoxification and preserve cytoskeletal integrity; zinc contributes to membrane stabilization and mechanosensitive signaling; and iron overload promotes ferroptosis, lipid peroxidation, endothelial stiffness, and extracellular matrix remodeling. Therefore, micronutrient imbalance may directly amplify the redox–mechanobiological feedback cycle described throughout this review.

However, despite growing evidence, several limitations remain. Most available studies are experimental and rely on isolated cell culture systems rather than physiologically relevant tissue models. Furthermore, methodologies used to assess cellular biomechanics—including atomic force microscopy, traction force microscopy, and microfluidic deformability assays—remain poorly standardized across studies.

Another important limitation is the lack of longitudinal clinical investigations directly correlating oxidative stress biomarkers with biomechanical measurements. Although endothelial stiffness and altered deformability have been observed in cardiovascular disease and COVID-19, causality remains incompletely established.

Conflicting findings also exist regarding antioxidant supplementation. While selenium and zinc supplementation may improve antioxidant capacity in selected populations, large-scale clinical trials have produced inconsistent therapeutic outcomes [49, 50]. This inconsistency likely reflects the multifactorial nature of mechanoredox signaling and suggests that antioxidant therapy alone may be insufficient without simultaneous modulation of biomechanical dysfunction.

## Conclusion

Despite substantial progress in understanding the redox–mechanobiological axis, several major controversies and unresolved questions remain.

First, it is still unclear whether oxidative stress is a primary initiator of biomechanical dysfunction or a secondary consequence of altered mechanical signaling. The temporal relationship between ROS generation and cytoskeletal remodeling has not been fully established in vivo.

Second, the role of antioxidant supplementation remains controversial. While selenium and zinc deficiency are associated with poor clinical outcomes in cardiovascular disease and COVID-19, interventional trials have yielded inconsistent results. Differences in baseline micronutrient status, disease stage, dosage, and bioavailability likely contribute to these discrepancies.

Third, the molecular mechanisms linking mechanotransduction pathways to mitochondrial redox signaling remain incompletely characterized. In particular, the interactions among YAP/TAZ activation, mitochondrial dynamics, and ferroptosis require further investigation.

Future research should focus on:

- Development of integrated mechanobiological and redox profiling approaches.
- Standardization of cellular stiffness and deformability measurements.
- Investigation of mitochondrial biomechanics in inflammatory disease.
- Exploration of ferroptosis as a mechanoredox pathway.
- Evaluation of combination therapies targeting both oxidative stress and biomechanical dysfunction.
- Translation of mechanobiological biomarkers into clinical diagnostics.

Advanced technologies including organ-on-chip systems, high-resolution live-cell imaging, multi-omics analysis, and artificial intelligence-assisted biomechanical modeling may substantially improve mechanistic understanding and therapeutic development.

Future translational studies integrating redox biology, mechanobiology, mitochondrial physiology, and trace element homeostasis may substantially improve understanding of disease progression and facilitate development of novel mechanoredox-targeted therapeutic strategies.

## Data Availability

No datasets were generated or analysed during the current study.
